# Diagnostic performance of ^18^F-fluorodeoxyglucose-PET/MRI versus MRI alone in the diagnosis of pelvic recurrence of rectal cancer

**DOI:** 10.1007/s00261-021-03224-3

**Published:** 2021-08-17

**Authors:** Verena Plodeck, Ivan Platzek, Johannes Streitzig, Heiner Nebelung, Sophia Blum, Jens-Peter Kühn, Ralf-Thorsten Hoffmann, Michael Laniado, Enrico Michler, Sebastian Hoberück, Klaus Zöphel, Jörg Kotzerke, Johannes Fritzmann, Jürgen Weitz, Christoph G. Radosa

**Affiliations:** 1 Department of Radiology, Dresden University Hospital, Fetscherstrasse 74, 01307 Dresden, Germany; 2 Department of Nuclear Medicine, Dresden University Hospital, Fetscherstrasse 74, 01307 Dresden, Germany; 3 Department of Nuclear Medicine, Helios Hospital Erfurt, Nordhäuser Strasse 74, 99089 Erfurt, Germany; 4 Department of Nuclear Medicine, Chemnitz Hospital GmbH, Flemmingstrasse 2, 09116 Chemnitz, Germany; 5 Department of General, Thoracic and Vascular Surgery, Dresden University Hospital, Fetscherstrasse 74, 01307 Dresden, Germany

**Keywords:** ^18^F-FDG-PET/MRI, MRI, Rectal cancer, Recurrence

## Abstract

**Purpose:**

To compare the diagnostic performance of ^18^F-fluorodeoxyglucose-PET/MRI and MRI in the diagnosis of pelvic recurrence of rectal cancer.

**Methods:**

All PET/MRIs of patients in the follow-up of rectal cancer performed between 2011 and 2018 at our institution were retrospectively reviewed. Recurrence was confirmed/excluded either by histopathology or imaging follow-up (> 4 months). Four groups of readers (groups 1/2: one radiologist each, groups 3/4: one radiologist/one nuclear medicine physician) independently interpreted MRI and PET/MRI. The likelihood of recurrence was scored on a 5-point-scale. Inter-reader agreement, sensitivity, specificity, PPV/NPV and accuracy were assessed. ROC curve analyses were performed.

**Results:**

Fourty-one PET/MRIs of 40 patients (mean 61 years ± 10.9; 11 women, 29 men) were included. Sensitivity of PET/MRI in detecting recurrence was 94%, specificity 88%, PPV/NPV 97% and 78%, accuracy 93%. Sensitivity of MRI was 88%, specificity 75%, PPV/NPV 94% and 60%, accuracy 85%. ROC curve analyses showed an AUC of 0.97 for PET/MRI and 0.92 for MRI, but the difference was not statistically significant (*p* = 0.116). On MRI more cases were scored as equivocal (12% versus 5%). Inter-reader agreement was substantial for PET/MRI and MRI (0.723 and 0.656, respectively).

**Conclusion:**

^18^F-FDG-PET/MRI and MRI are accurate in the diagnosis of locally recurrent rectal cancer. Sensitivity, specificity, PPV, NPV and accuracy are comparable for both modalities, but PET/MRI increases readers’ confidence levels and reduces the number of equivocal cases.

**Graphic abstract:**

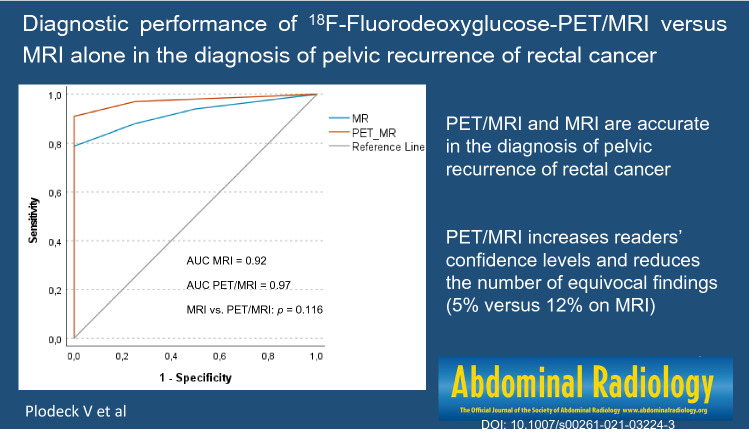

## Introduction

Colorectal cancer (CRC) is the third most common cancer worldwide, accounting for approximately 10% of newly diagnosed cancers and 9% of cancer deaths [1]. It is expected that numbers will increase by around 60% to more than 2.2 million new cases by 2030 [2]. Rectal cancer accounts for about 40% of all cases of CRC [1].

Depending on tumor stage, surgery alone or (chemo-)radiotherapy combined with surgery is the mainstay of curative treatment in rectal cancer. Local recurrence rates have decreased significantly after the introduction of total mesorectal excision and neoadjuvant radiotherapy, now ranging between 5 and 10% [3, 4]. In the case of local recurrence, complete surgical resection is the only curative treatment and has been shown to improve survival [5–7]. The diagnosis can be difficult, though, due to pelvic scarring and inflammatory/reactive changes after radiotherapy or anastomotic leaks [8–11]. Furthermore, complete resection is challenging and due to the potential extent up to pelvic exenteration, accurate preoperative diagnosis is essential [12–14].

Currently CT is the most commonly used modality in the follow-up of rectal cancer. A review by Schaefer et al. described the sensitivity of CT for diagnosing pelvic recurrence around 80%, with specificity ranging from 50 to 97% [15–17]. Due to better differentiation between scar tissue and tumor recurrence, MRI yields higher sensitivity (87–100%), whereas specificity is comparable to CT (48–91%) [18–20]. Several studies have investigated the use of ^18^F-FDG-PET in this setting, with accuracy ranging from 74 to 96% and sensitivity and specificity of 84% and 88%, respectively [15, 21]. PET/CT was shown to facilitate the differentiation between benign and malignant presacral lesions (sensitivity 100%, specificity 96%) [8]. However, a recent study described a PPV of only 58% for FDG-avid presacral lesions on PET/CT, but improved diagnostic performance after the addition of MRI sequences [22]. PET has also been shown to be reliable in the detection of metastases, as well as having significant impact on further management [23, 24]. The use of PET has been recommended in equivocal cases and prior to extensive resection [23].

MRI, PET and PET/CT have been described as accurate in the diagnosis of locally recurrent rectal cancer. The combination of functional imaging with PET and the excellent soft tissue contrast of MRI could prove useful in this setting. In a recent study, which has so far, to the best of our knowledge, been the only work on PET/MRI in pelvic recurrence of rectal cancer, PET/MRI demonstrated good accuracy (94%) [25]. This retrospective, single-reader study assessed the diagnostic performance of PET/MRI without comparison to MRI or other imaging modalities.

Thus, our aim was to compare ^18^F-FDG-PET/MRI and MRI in the diagnosis of pelvic recurrence of rectal cancer.

## Materials and methods

### Patients

The local ethics committee granted approval for this study. Patients had given written informed consent to PET/MRI, as well as review of their records and imaging studies for scientific purposes. Clinical data and imaging of 69 consecutive patients (23 women, 46 men) who had received PET/MRI in the follow-up of rectal cancer at our institution between 2011 and 2018 were retrospectively reviewed and screened for eligibility. 18 patients received two PET/MRIs, thus a total of 87 PET/MRIs was reviewed.

### ^18^F-FDG-PET/MRI

Preparation followed our routine protocol. Patients were asked to fast (≥ 6 h) before the examination and blood glucose levels were measured (4.6–11.9 mmol/l, median 5.7 mmol/l). 4.5 MBq ^18^F-FDG/kg body weight was administered intravenously (277–377 MBq, median 313 MBq; GlucoRos®, Helmholtz-Zentrum Dresden-Rossendorf, Germany). The time between ^18^F-FDG injection and PET/MRI was 58–128 min (median 74 min).

PET/MRI was performed on a 3 T scanner with patients in supine position, arms by the sides (Ingenuity TF PET/MR; Philips Health Systems, Amsterdam, Netherlands). Nine to ten bed positions with an overlap of 9 cm were acquired with a scan time of 2 min each. Field-of-view was 18 cm, reconstructed isotropic spatial resolution 5.5 cm. Low-resolution T1-weighted fast-field-echo images were obtained from head to distal femur (integrated quadrature body coil) to create a map for attenuation correction via segmentation into three tissue classes (air, lung, soft tissue), followed by an assignment of respective attenuation values. Patient’s position remained unchanged to achieve optimal co-registration and PET was performed immediately after the attenuation scan. If tracer in the urinary bladder was thought to mask recurrence, another pelvic PET scan was performed after voiding.

MRI was performed according to our standard protocol for pelvic MRI in the follow-up of pelvic malignancy. Since most recurrences occur extraluminally [6] and the majority of our cohort had not undergone continence-preserving surgery, this protocol was chosen rather than using a dedicated MRI protocol for primary rectal cancer. Thus, all pelvic MRIs included T2-weighted imaging, diffusion-weighted imaging (DWI) and T1-weighted contrast-enhanced (CE) sequences (Sense-Xl-Torso coil, for details see Table 1). Apparent diffusion coefficient (ADC) maps were generated automatically.

**Table 1:** MRI parameters

	T2 TSE axial	T2 TSE sagittal	DWI axial^a^	T1 TSE CE axial^b^
FOV (mm)	270 × 318	240 × 240	375 × 375	270 × 318
TE (ms)	100	100	52	10
TR (ms)	4463	3626	1825	660
FA	90	90	90	90
Matrix	672 × 672	320 × 320	256 × 256	400 × 400
Slice thickness (mm)	5	4	5	5
Slice gap (mm)	5.5	4	5	6
Turbo factor (TE, ms)	100	100	53	10
Fat suppression	No	No	Yes	Yes
NSA	2	2	7	2
Voxel size (mm)	0.49	0.78	1.46	0.82
Acquisition time (min)	2.45	3:26	4:15	2:50
EPI factor			57	

*TSE* turbo spin echo, *DWI* diffusion-weighted imaging, *CE* contrast enhanced, *FOV* field of view, *TE* echo time, *TR* repetition time, *FA* flip angle, *NSA* number of signals averaged

^a^DWI with *b*-values of 0, 100, 800

^b^Contrast-enhanced sequences were performed about 60 s after the intravenous administration of 0.2 ml gadolinium diethylenetriamine penta-acetic acid or 0.1 ml gadobutrol per kg body weight (Magnevist®/Gadovist®, Bayer Pharma, Berlin, Germany), followed by 20 ml Saline

Fused PET/MR images including multiplanar reconstructions were created using the Philips Fusion Viewer software.

### Readings

Images were reviewed at a PACS workstation (AGFA Healthcare, Impax EE R20 XVIII, Bonn, Germany). Four groups of readers were formed. All readers had more than 5 years of experience in MR and/or hybrid imaging. The first two consisted of one radiologist each (JS/CGR), who independently reviewed all MRIs for pelvic recurrence. Then one radiologist and one nuclear medicine physician were assigned to each other to build two more groups (JS/EM; CGR/SH). To avoid bias a time gap of 3 months was kept between the readings. PET/MRIs were jointly reviewed by the nuclear medicine physician and the radiologist. Each finding was scored 0–4 (0 = no recurrence, 1 = recurrence unlikely, 2 = equivocal, 3 = probable recurrence, 4 = definite recurrence).

To achieve a final reading, expert readers with more than 15 years of experience (MRI: one radiologist JPK, PET/MRI: one radiologist/one nuclear medicine physician IP/KZ) reviewed all cases, where readings differed between the MRI groups or between the two PET/MRI groups and chose the final score out of the two differing scores. Readers were blinded to medical history including primary tumor stage and histology, prior imaging, referral diagnoses and each other’s results.

MRI criteria for malignancy were: On T2, nodular or irregularly shaped soft tissue masses, inhomogeneous structure, signal intensities equivalent or higher compared to muscle and/or infiltration of adjacent organs. On DWI high signal on b800 images with corresponding low signal on ADC maps and on CE MRI inhomogeneous enhancement with suspicious morphology on T2.

PET/MRI criteria for pelvic recurrence considered shape, location and intensity of ^18^F-FDG uptake. No threshold standardized uptake value (SUV) has been established for pelvic recurrence of rectal cancer, therefore the diagnosis was based on visual assessment. Increased uptake compared to liver background was regarded as suspicious [26]. ^18^F-FDG uptake lower than liver background in anatomical structures like ovaries or rectal stump and typical uptake in the urinary tract or along bowel loops was considered benign [8, 27]. Photopenic areas with slight uptake of the margins were considered fluid collections. Non-focal uptake, less compared to background, was presumed to be due to inflammation, if this corresponded to MRI. In case of differing assessments on PET and MRI, a consensus had to be reached in each case.

Lesions were considered malignant if they showed intense focal ^18^F-FDG uptake, as well as suspicious MR findings as described above. No recurrence was diagnosed if there was neither focal uptake on PET, nor suspicious MR findings. According to the readers’ confidence levels scores 3 and 4 or 0 and 1 were chosen, when both readers agreed on the presence or absence of local recurrence. Score 2 was used when both readers were unsure, if pelvic recurrence was present.

### Statistical analysis

Imaging was considered true-negative, if no tumor was found at histopathology or if the lesion remained unchanged or reduced in size without treatment. Findings were considered true-positive, if a suspicious lesion was confirmed on histopathology or showed progression over follow-up of at least 4 months.

Sensitivity, specificity, PPV/NPV and accuracy were determined using lesion-based analyses and a contingency table. Since equivocal findings lead to further investigations in clinical practice, scores were dichotomised into imaging negative (score 0 or 1) and imaging positive groups (scores 2, 3 or 4) to calculate diagnostic performance. Interrater agreement was determined using weighted Cohen’s Kappa with linear weights. To compare readings ROC curve analyses were performed and AUCs were calculated (CGR/HN). The significance level was set at *α* = 0.05 (SPSS for Windows, SPSS Statistics 27, IBM, Armonk, US).

## Results

**Fig. 1:**
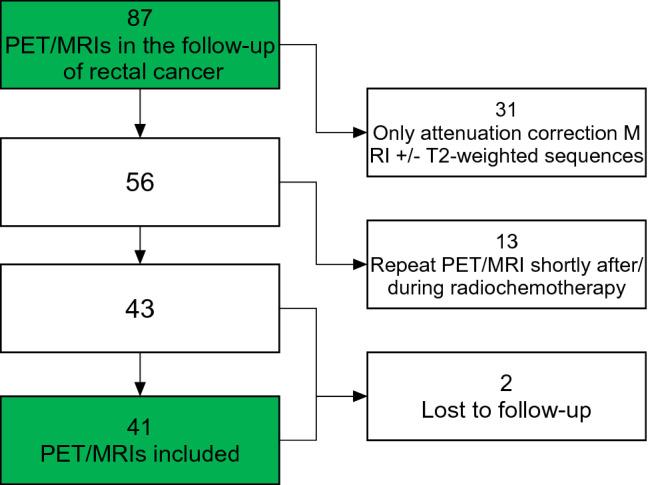
Exclusion criteria

Out of 87 PET/MRIs 31 had to be excluded because they only included a low-resolution attenuation MRI ± T2-weighted sequences. These patients were mainly examined shortly after we established PET/MRI in this setting in 2011 and before we adjusted the protocol to include a full diagnostic MR scan. Since our goal was to assess the diagnostic performance in the primary diagnosis of recurrence, another 13 repeat PET/MRIs for re-staging during or shortly after chemoradiotherapy (< 3 months) were excluded. Two patients were lost to follow-up, the remaining 41 PET/MRIs were included (see Fig. 1, ClickCharts®, NCH Software, Inc., Greenwood Village CO, USA). One repeat PET/MRI was included, the patient had undergone resection of pelvic recurrence and then received PET/MRI at a later time point for equivocal findings on previous other imaging.

Thus, we included 40 patients with a mean age of 61 years (± 10.9), 29 were men, 11 women. Primary treatment of rectal cancer was abdominoperineal resection (19 patients), anterior resection (17 patients), restorative coloproctectomy (two patients), full thickness local excision and curative chemoradiotherapy (one patient each). The majority of patients had received neoadjuvant therapy (27/40). Further patient characteristics are summarized in Table 2.

**Table 2:** Patient characteristics

Age	33–77 years (mean 61 years)
Gender	
Male	29
Female	11
Primary tumor stage (UICC)	
Stage I	8
Stage IIa	10
Stage IIb	1
Stage IIIa	2
Stage IIIb	10
Stage IIIc	4
Stage IV	2
Unknown	3
Primary R state	
R0	28
R1	7
R2	0
Rx	2
Unknown	3
Primary tumor histology	
Adenocarcinoma	29
Mucinous adenocarcinoma	4
Unknown	7
Previous therapy	
Abdominoperineal resection	19
Neoadjuvant radiochemotherapy	12
Neoadjuvant radiotherapy	0
Neoadjuvant chemotherapy	1
No neoadjuvant therapy	5
Unknown	1
Anterior resection	17
Neoadjuvant chemoradiation	12
Neoadjuvant radiotherapy	1
Neoadjuvant chemotherapy	1
No neoadjuvant therapy	3
Restorative coloproctectomy	2
Full thickness local excision	1
Curative chemoradiation	1

*UICC* Union for International Cancer Control

Indications for PET/MRI were equivocal findings on previous other imaging (23/41), follow-up after rectal cancer (4/41), clinical findings suspicious of recurrence (1/41) and staging of proven recurrence (13/41). Median SUV of suspicious lesions was 6.3 (range 3.9–26). Size of local recurrences ranged from 1.1 to 11 cm (median 4.2 cm). All 33 pelvic recurrences were single-site and proven by histopathology. The eight cases without recurrence were confirmed by follow-up, one also had negative biopsies. Follow-up ranged between 16 and 208 weeks (median 72 weeks) and included CT and MRI as well as clinical findings. 24 patients underwent curative resection of the recurrence. After resection of the recurrence 66.7% (16/24) of patients had histologically negative resection margins (R0), 12.5% (3/24) were classified as microscopically residual tumor (R1), another 20.8% (5/24) as uncertain resection margins (Rx).

**Fig. 2:**
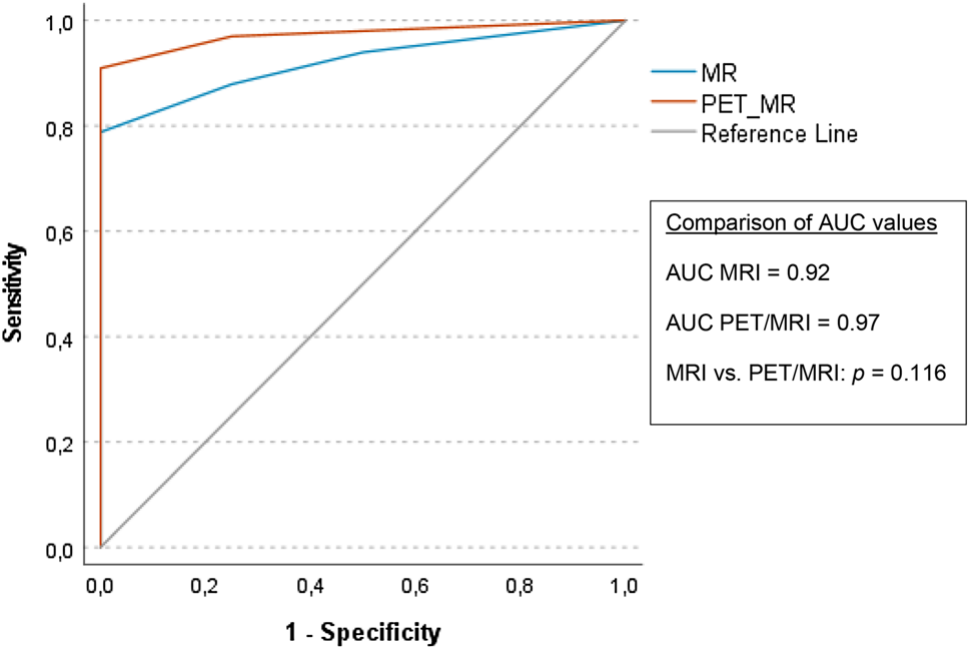
ROC curves and AUCs

Sensitivity of ^18^F-FDG-PET/MRI in detecting recurrence was 94% (31/33), specificity 88% (7/8), positive predictive value 97% (31/32), negative predictive value 78% (7/9) and accuracy 93%. Sensitivity of MRI was 88% (29/33), specificity 75% (6/8), positive predictive value 94% (29/31), negative predictive value 60% (6/10) and accuracy 85%. ROC curve analyses showed an AUC of 0.97 for PET/MRI and 0.92 for MRI, but the difference was not statistically significant (*p* = 0.116), see also Fig. 2 and Table 3.

**Table 3:** Diagnostic performance

	PET/MRI	MRI
Sensitivity	94% (31/33)	88% (29/33)
95% CI	86–100%	77–99%
Specificity	88% (7/8)	75% (6/8)
95% CI	65–100%	45–100%
PPV	97% (31/32)	94% (29/31)
95% CI	91–100%	85–100%
NPV	78% (7/9)	60% (6/10)
95% CI	51–100%	30–90%
Accuracy	93% (38/41)	85% (35/41)
95% CI	85–100%	75–96%
AUC	0.97	0.92
95% CI	0.93–1.00	0.84–1.00

*PPV* positive predictive value, *NPV* negative predictive value, *AUC* area under the ROC curve, *CI* confidence interval

**Fig. 3:**
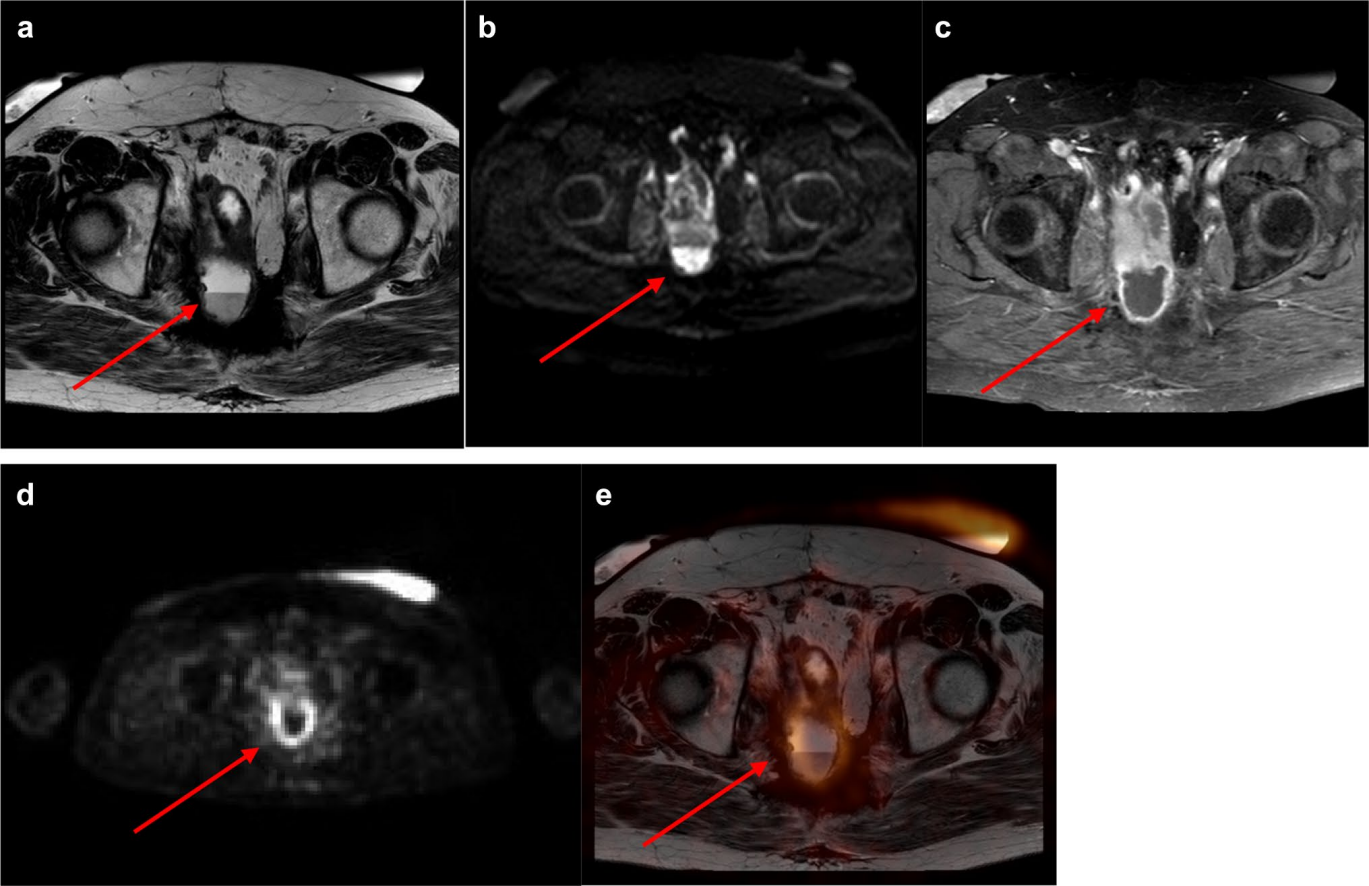
**a**–**d** Chronic inflammation with presacral fluid collection. **a**–**c** Axial T2-weighted/axial diffusion-weighted/axial T1-weighted contrast-enhanced MRI. MRI showing a presacral, partially T2-hyperintense lesion with a thick hypointense rim and partially restricted diffusion, fluid-level and contrast enhancement of the rim. MRI was scored as equivocal (score 2). **d**/**e** Corresponding PET and PET/MRI fusion images demonstrating slightly increased FDG-uptake presacrally. PET/MRI was scored as equivocal (score 2). Follow-up of more than 4 years excluded tumor recurrence

On PET/MRI one case without recurrence was scored as equivocal and thus considered as false-positive. The patient suffered from familial adenomatous polyposis and had undergone chemoradiation and pelvic exenteration for mucinous adenocarcinoma. Consequently, the patient developed chronic presacral fluid collections and fistulas (Fig. 3). Follow-up of 4 years excluded recurrence. PET/MRI also missed two recurrences, adjacent to the right ureter and to small bowel loops, respectively.

**Fig. 4:**
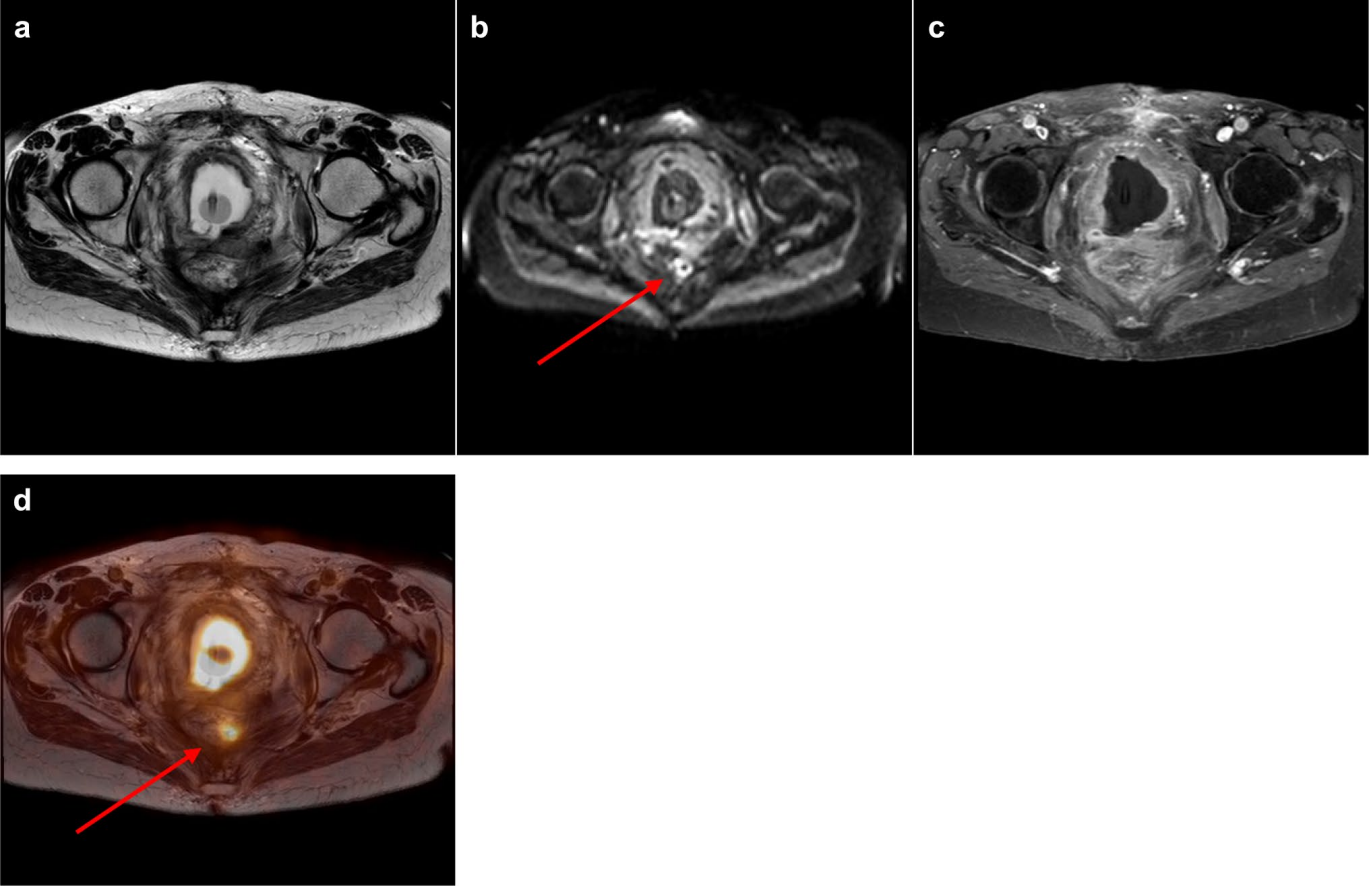
**a**–**d** Small presacral tumor recurrence. **a**–**c** Axial T2-weighted/axial diffusion-weighted/axial T1-weighted contrast-enhanced MRI. MRI showing diffuse T2-hypointense changes presacrally, a small rim-like diffusion restriction and no focal contrast enhancement. Diffusion restriction was thought to be due to reactive/inflammatory changes. MRI was scored as recurrence unlikely (score 1). **d** Corresponding axial PET/MRI fusion image demonstrating a small presacral lesion with intense FDG-uptake, which was interpreted as highly suspicious (score 4) for tumor recurrence. Biopsy confirmed tumor recurrence

When classifying only scores 0 (instead of 0/1) and 4 (instead of 2/3/4) as correctly negative or positive, on PET/MRI 76% (25/33) of recurrences were diagnosed correctly, on MRI 70% (23/33), PET/MRI excluded recurrence correctly in 75% (6/8), MRI in 50% (4/8). MRI classified more cases as equivocal compared to PET/MRI (5/41 versus 2/41, 12% versus 5%), for an example see Fig. 4.

Inter-reader (for PET/MRI inter-groups of readers) agreement was substantial for MRI and PET/MRI (for MRI 0.656, 95% confidence interval 0.474–0.838; PET/MRI 0.723, 95% confidence interval 0.554–0.892).

## Discussion

The detection of pelvic recurrence of rectal cancer is challenging, but accurate diagnosis is very important due to the potential extent of resection, which remains the only potentially curative treatment. Our study shows that compared to MRI alone, PET/MRI leads to less equivocal findings (5% versus 12%) and increases readers’ confidence in diagnosing or excluding local recurrence. Inter-reader agreement (0.723 versus 0.656), sensitivity (94% versus 88%), specificity (88% versus 75%), PPV/NPV (97/94% versus 78/60%) and accuracy (93% versus 85%) are comparable for MRI and PET/MRI in the diagnosis of locally recurrent rectal cancer. PET/RMI showed higher AUC (0.97 versus 0.92), but the difference was not statistically significant (*p* = 0.116).

MRI has been studied extensively in this setting and has shown promising results, with sensitivity of up to 90%, but variable specificity [15, 18, 20]. A study by Lambregts et al. included 42 patients with the clinical suspicion of recurrence and found good accuracy (79–95%, depending on the experience of the reader and the sequences reviewed) of standard T2-weighted MRI and T2 + DWI [19]. We found similar accuracy, sensitivity and PPV as the less experienced reader, but could not reach the expert reader’s diagnostic performance. Specificity and NPV were lower in our study. These differences could be due to the low number of disease negative patients in our study as well as the intermediate level of experience of both our MRI readers. Furthermore, readers in the study by Lambregts et al. were not blinded to treatment, histological stage or tumour type of the primary rectal cancer and 9/42 patients were classified as R1 or R2 on histopathology after the primary resection, which might have introduced some bias into the readings. No patients after abdominoperineal resection (18/40 in our study) were included, which might also have contributed to our partially differing results. Molinelli et al. investigated the added value of DWI and CE MRI in patients with suspected local recurrence [20]. They concluded that adding DWI and CE sequences improved diagnostic performance and inter-reader agreement. AUCs in this study for T2 + contrast-enhanced T1 + DWI ranged between 0.960 and 0.999, depending on the experience level of the readers. No patients with mucinous adenocarcinoma (5/40 in our study) were included in this study, which could be an explanation for the lower diagnostic performance of MRI in our study, since recurrence of mucinous tumors has been shown to cause false negative results on diffusion-weighted MRI [28].

PET and PET/CT have been described in the literature as reliable methods in the diagnosis of pelvic recurrence of rectal cancer as well as the detection of metastases, thus having significant impact on further management [8, 21, 23, 24]. PET/MRI combines functional imaging and excellent soft tissue contrast and could therefore be very useful in patients with tumor recurrence. A recent retrospective, single-reader study on PET/MRI reported a sensitivity of 94%, specificity of 94%, PPV 97%, NPV 90% and accuracy of 94% for the detection of local recurrence [25]. Our study showed comparable sensitivity, PPV and accuracy, but lower specificity and NPV. This is likely due to the relatively low number of cases without recurrence and the statistical cut-off at scores > 1, which led to equivocal cases being considered as recurrences. The false-positive case on PET/MRI showed inflammatory changes and was scored as equivocal, which would have been less likely, if readers had not been blinded to previous history and other findings. The two false negative cases were both recurrences adjacent to structures with physiological tracer uptake or excretion, which is a common pitfall on PET/MRI [26]. PET/MRI involves patient exposure to ionizing radiation, which needs to be taken into account. Due to potentially extensive surgery in pelvic recurrence of rectal cancer and the aim to achieve R0 resection in this group of patients, the benefit of even a slight improvement in diagnostic accuracy could outweigh this disadvantage.

There are some limitations to our study. Due to the high number of recurrences found, a selection bias is likely and the low number of patients without recurrence leads to large confidence intervals on statistical analysis. We tried to counter this limitation by reviewing all consecutive PET/MRIs of patients with a history of rectal cancer, but with PET/MRI being used almost exclusively as a second-line investigation and not in the standard follow-up, the majority of patients showed recurrence. Further limitations are the relatively low overall number of cases and the retrospective study design.

In conclusion, PET/MRI and MRI are accurate in the diagnosis of pelvic recurrence of rectal cancer. PET/MRI increases readers’ confidence levels and reduces the number of equivocal findings compared to MRI alone. Thus, we suggest using PET/MRI in patients with equivocal findings in other imaging modalities, particularly in a preoperative setting.

## Data Availability

The datasets generated and/or analysed are available from the corresponding author on reasonable request.

## Abbreviations

FDG: Fluorodeoxyglucose
PPV: Positive predictive value
NPV: Negative predictive value
ROC: Receiver operator characteristic
AUC: Area under the curve
CRC: Colorectal cancer
DWI: Diffusion-weighted imaging
CE: Contrast-enhanced
SUV: Standardized uptake value
